# Wine Phenolic Compounds: Chemistry, Functionality and Health Benefits

**DOI:** 10.3390/antiox13111312

**Published:** 2024-10-28

**Authors:** Youssef El Rayess, Nancy Nehme, Samar Azzi-Achkouty, Sofi G. Julien

**Affiliations:** 1 Department of Agriculture and Food Engineering, School of Engineering, Holy Spirit University of Kaslik, Jounieh P.O. Box 446, Lebanon; samarazzi@usek.edu.lb; 2 Faculty of Agricultural Engineering and Veterinary Medicine, Lebanese University, Dekwaneh P.O. Box 446, Lebanon; nancy.nehme@ul.edu.lb; 3 Department of Nutrition and Food Sciences, Faculty of Art and Sciences, Holy Spirit University of Kaslik, Jounieh P.O. Box 446, Lebanon

**Keywords:** wine polyphenols, flavonoids, antioxidant, bioactivity, non-communicable diseases, in vivo studies, in vitro studies, human health

## Abstract

Wine phenolic compounds, often known as polyphenols, are a diverse group of secondary bioactive compounds derived from grapes. They play a crucial role in defining the sensory characteristics, functionality, and health benefits of wine. This review explores the complex chemistry of these compounds, focusing on key classes such as flavonoids, which include flavanones, flavonols, anthocyanins, and flavan-3-ols, and non-flavonoids, such as hydroxycinnamic acids, hydroxybenzoic acids, and stilbenes. The health benefits of wine phenolics, particularly their antioxidant and anti-inflammatory properties, are also discussed in relation to preventing and reducing the risk of non-communicable diseases (NCDs) such as cardiovascular diseases, cancers, and neurodegenerative conditions. Furthermore, this review summarized the most current data from human population-based research that investigated the bioactivity of these red wine phytochemicals with relevant health benefits for NCDs. Finally, this review proposes some perspectives for future research to better understand the bioavailability, metabolism, and long-term health effects of these compounds.

## 1. Introduction

Due to the alarming rise in the prevalence of non-communicable diseases (NCDs) worldwide, which is primarily attributable to sedentary behavior and poor dietary habits, researchers have concentrated on the nutritional management of these conditions. They have developed a diverse array of diets that are either more or less effective [1]. A prevalent determinant in the etiology of these disorders is oxidative stress. Recently, research in this field has made significant progress in comprehending the synergistic relationship between the intake of natural food compounds and their utilization as biologically active molecules following their physiological processing through digestive, absorptive, metabolic, or fermentation reactions. It is imperative that their bioactivity be demonstrated through their proper bioavailability and utilization by body cells and tissues.

The ability of a substance to function as an electron donor and prevent oxidative degeneration is a chemical property known as an antioxidant. Reactive oxygen species (ROS) are implicated in the development of age-related and NCDs, as documented by numerous studies [2]. Dietary antioxidants have been proven to effectively combat these conditions [3]. Phenolic compounds are the primary antioxidants found in food, particularly in wines, tea, chocolate, fruits, and liquids.

The potential of polyphenols as free-radical scavengers or antioxidants is predicted by their chemical activities. Four factors determine the activity of an antioxidant: (i) its reactivity as a hydrogen or electron-donating agent (which is related to its reduction potential); (ii) the fate of the resulting antioxidant-derived radical, which is regulated by its capacity to stabilize and delocalize the unpaired electron; (iii) its reactivity with other antioxidants; and (iv) the transition metal-chelating potential [4,5].

Wine is a wealthy source of polyphenols, which are responsible for a variety of technological and quality attributes. Technological properties encompass the physicochemical stability of wines and the capacity of wine-aging attitudes, while they contribute to the color, hue, clarity, flavor, bitterness, and astringency of a wine [6,7].

Polyphenols are secondary metabolites that vines produce in response to adverse conditions during normal development [8,9]. Several factors influence the biosynthesis and accumulation of polyphenols through the general phenylpropanoid pathway during berry maturation. They include the cultivar varieties (clones and rootstock), the environmental factors (agro-pedological, topographical, and climatic), and the cultural practices (training systems, row vine spacing, pruning, bunch reduction, bud and leaf removal, as well as water, fertilizers, and pesticide management). For these reasons, the polyphenolic composition of the wine differs from that of the grape. The polyphenols are indeed diffused and extracted through maceration during the winemaking process, with the formation of the must serving as the initial step in the process that culminates in the production of wine in subsequent phases. This process necessitates numerous chemical modifications and interactions with other molecules.

Due to the importance and attention paid to the literature on polyphenols in human health and wine quality, it is necessary to have well-developed, reliable, and established methods to identify and measure wine polyphenols as well as to assess their antioxidant potential.

The purpose of this review is to recollect the chemistry of wine polyphenols and their health-promoting properties to summarize the current knowledge and identify voids and areas for future research. 

## 2. The Chemistry of Wine Phenolic Compounds

**Figure 1:**
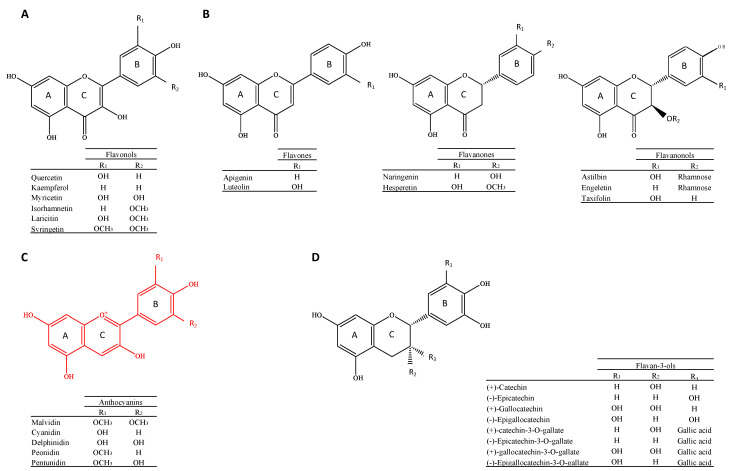
Basic chemical structure of the subfamily of flavonoids. R_1_, R_2_ and R_3_ represent the substituents on the different wine phenolic flavonoid compounds: Flavonols (**A**); Flavones, Flavanones and Flavanonols (**B**); Anthocyanins (**C**) and Flavan-3-ols (**D**). Flavanones. General structure of flavnoids consist of benzene rings A and B linked by a heterocyclic pyrane ring.

**Figure 2:**
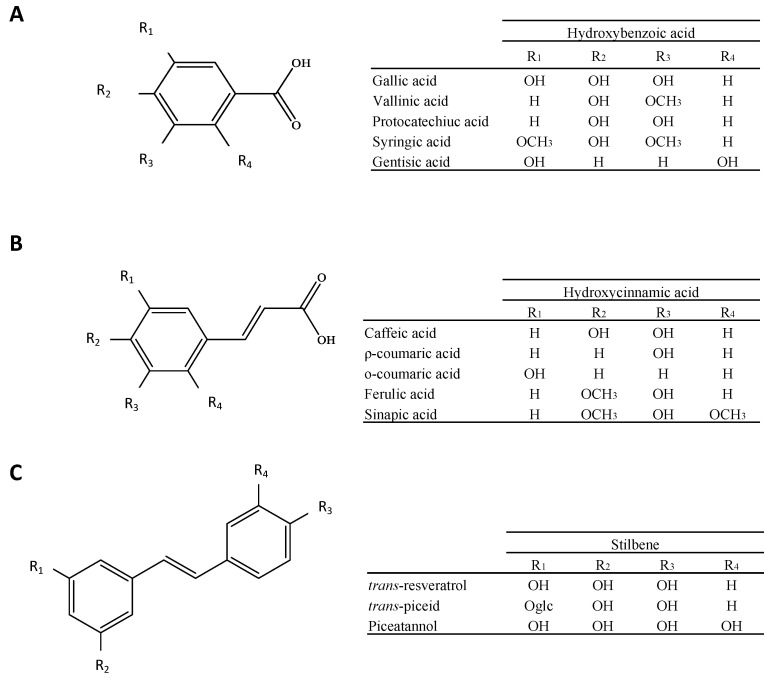
Basic chemical structure of the subfamily of non-flavonoids. R_1_, R_2,_ R_3_ and R_4_ represent the substituents on the different subfamily non-flavonoid compounds: Hydroxybenzoic acid (**A**); Hydroxycinnamic acid (**B**) and Stilbene (**C**).

The structural diversity of wine phenolic compounds is extensive, reflecting the wide array of chemical constituents present in grapes and their transformation during winemaking. Polyphenols in general are very reactive and unstable chemical structures that can evolve over time and depend on many factors. Therefore, the phenolic compound profile in wine is quite different than that found in grapes. The chemical processes responsible for these changes have been extensively reviewed [10,11,12]. Phenolic compounds in wine can be broadly classified into two main groups: flavonoids and non-flavonoids. The flavonoids (Figure 1) are based on a common C6-C3-C6 skeleton; the non-flavonoids include C6-C1 hydroxybenzoic acids, C6-C3 hydroxycinnamic acids, and C6-C3-C6 stilbenes (Figure 2). 

### 2.1. Flavonoids

The flavonoids’ basic skeleton consists of 15 carbons linked by a three-carbon chain cyclized through oxygen. Grape and wine flavonoids have a hydroxyl group in positions 5 and 7 of the A ring. Flavonoids cover a large number of subclasses, such as flavonols (Figure 1A); flavones, flavanones, and flavanonols (Figure 1B); anthocyanins (Figure 1C); and flavan-3-ols (Figure 1D).

#### 2.1.1. Flavonols

Flavonols are yellow pigments located in grape berry skins. They can be found in white and red wines. They contribute to the color of white wine while they are involved in the copigmentation phenomenon in red wine [13,14]. Flavonol biosynthesis has been shown to be under genetic control and to be light-dependent [15]. Their chemical structure is characterized by the existence of a ketone group at position 4 and a hydroxyl group at position of the C ring (Figure 1A). Flavonols can have different substitutions on the A and B rings, including hydroxyl and methoxy groups, leading to structural diversity. Several similar flavonols have been detected and quantified in red and white wines [16]. The six major flavonols identified in the red grape cultivars according to the B-ring substitution pattern are kaempferol, quercetin, myricetin, isorhamnetin, laricitrin, and syringetin [17]. Rutin, also known as quercetin 3-O-rutinoside, is not considered a major grape flavonol. In grapes, flavonols are presented exclusively as glycosylated (3-O-glucosides, 3-O-galactosides, and 3-O-glucuronides), while in wines, free aglycones can be found together with the glycosylated form [18]. Free aglycones are released from the glycosylated ones due to hydrolysis (enzymatic or acidic) during winemaking. Apparently, the degree of hydrolysis is dependent on the flavonol structure and the nature of 3-O-glycoside. According to Makris et al. [19], the aglycones form of flavonols are labile molecules and may be degraded upon exposure to heat, enzymes, and oxidative chemical species. Therefore, grapes and wine processing and treatments might afford prominent alteration in the flavonol profile. On another hand, Favre et al. [20] demonstrated the occurrence of acetylated and p-coumaroylated derivatives of the flavonol-glucosides made from the methoxylated aglycones isorhamnetin, laricitrin, and syringetin in grapes and their respective wines. Red wine can contain up to 45 mg/L of flavonols [21]. Quercetin and its conjugated derivatives are frequently the main flavonols in both red and white grapes.

#### 2.1.2. Flavones, Flavanones, and Flavanonols

Flavones, flavanones, and flavanonols (dihydroflavonols) are rarely reported in wines. Flavone is a class of flavonoids based on the backbone of 2-phenylchromen-4-one and has three functional groups, including hydroxy, carbonyl, and conjugated double bond. Flavones are colorless-to-yellow crystalline substances [22]. The two detected flavones in wines are apigenin (0.2 mg/L) [23] and luteolin (0.2–7.2 mg/L) [24]. Flavanones have a saturated heterocyclic C ring with no conjugation between the A and B rings. The most representative compound of this family is naringenin (0.1–19.8 mg/L) [23,25]. The presence of naringenin is conditioned by the yeast strain used in alcoholic fermentation and the barrel aging process of wines. The most representative flavanonols in wines are astilbin (0.65–41.1 mg/L in red wines) [26], engeletin, and taxifolin (0.65–9.6) [27]. Astilbin is known to contribute to the sweet taste of wines [26,28,29], and its concentrations in wines are grape variety-dependent. The chemical structure of flavones, flavanones, and flavanonols and their respective functional groups are shown in Figure 1B. 

#### 2.1.3. Anthocyanins

**Figure 3:**
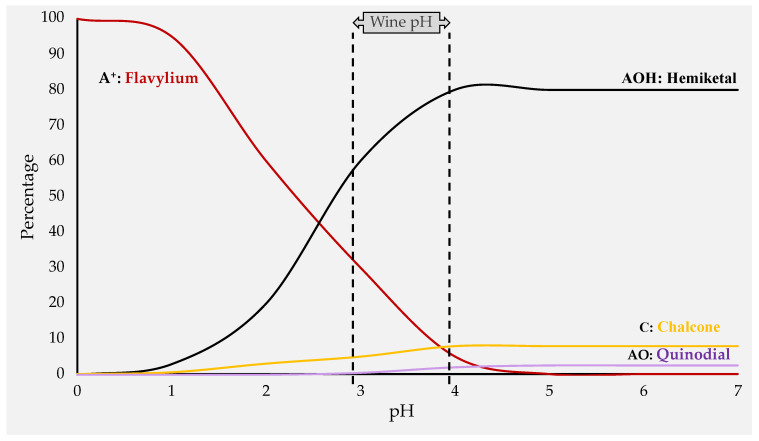
The pH-dependent equilibrium of anthocyanin structures that are present in wines.

The red color of grapes and wines is attributed to anthocyanins, which compose the largest group of pigments in the vegetal kingdom. These molecules are primarily found in the vacuoles of grape skin cells and occasionally in the flesh, particularly for teinturier varieties. All anthocyanins, which are also referred to as anthocyanidins, possess the aglycon form in their basic form. The primary anthocyanidins found in red wine are malvidin, petunidin, cyanidin, peonidin, delphinidin, and pelargonidin. These anthocyanidins are differentiated by the number and position of the hydroxyl and methoxyl groups on the B ring of the molecule (Figure 1C). Anthocyanins are the 3-O-monoglucoside form of anthocyanidins, which are particularly prevalent in wines produced from Vitis vinifera. In addition, the anthocyanidin-3,5-O-diglucosides, which were derived from Vitis riparia and Vitis rupestris, were identified using sensitive analytical techniques, particularly HPLC/MS [30]. However, the transition from grapes to wine alters the anthocyanin profile. Apart from the glucoside form of anthocyanins, acetic, lactic, p-coumaric, and caffeic acids esterify the glucose molecule at the C6 position, resulting in the acylated form [31,32]. Wine contains anthocyanins in four molecular forms known as flavylium, chalcone, hemiketal, and quinodal, all of which are in equilibrium. Figure 3 shows that the pH of the wine has a significant influence on their relative predominance in determining the wine color.

The red and purple pigments are influenced by the red flavylium cation (A^+^), which decreases as the pH increases. The flavylium form (A^+^) and the colorless hemiketal form (AOH) are the predominant forms at the wine pH level, typically falling between 3.3 and 3.6. The purple quinoidal base (AO) and the yellowish chalcone (C) forms are present at pH levels greater than 3.7. Proton transfers, hydration reactions, and tautomerism regulate the equilibrium between anthocyanin forms for the pairs AO/A^+^, A^+^/AOH, and AOH/C, respectively. The equilibrium of each of these pairs is determined by the dissociation constants: pKa = pH + log [A^+^]/[AO], pKh = pH + log [A^+^]/[AOH], and pKt = pH + log [C]/[AOH]. In addition to pH, the temperature and amount of free sulfur dioxide also influence the presence of these forms. While high temperatures may lead to a breakdown of the carbon chain and thus degradation of the pigments, high amounts of free sulfur dioxide can strongly bleach the free anthocyanins.

The composition of wine anthocyanin is contingent upon the original grape profile as well as the extraction and winemaking techniques employed. Red wine contains anthocyanin concentrations that vary between 20 and 500 mg/L [33]. Their concentrations are at their maximum during the initial days of fermentation. Subsequently, their quantities diminish due to their adhesion to yeast cell walls, their production of colloidal precipitates with tartaric salts, their removal during filtration and fining, and their involvement in numerous chemical reactions [6,34]. The anthocyanin pigments are destabilized by wine maturation, and their concentrations decrease as the wine matures. Conversely, physicochemical processes such as intramolecular and intermolecular copigmentations and self-association, which are known to be dependent on several factors, such as acidity, temperature, oxygen, and winemaking techniques [35,36], can stabilize various types of anthocyanins and their colors. These occur when anthocyanins interact hydrophobically with one another or with other compounds [37].

**Figure 4:**
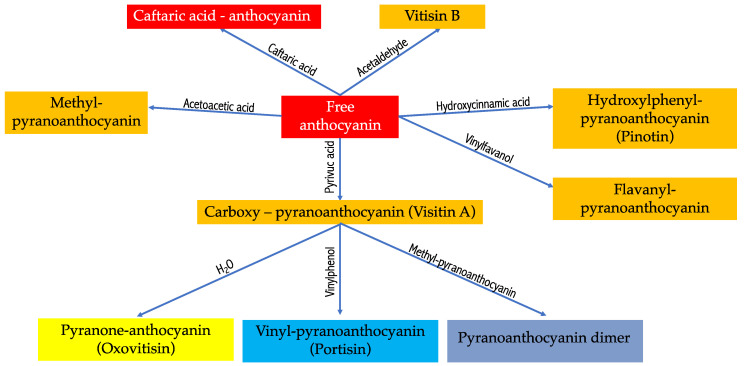
Cycloaddition reactions of free anthocyanins in red wines.

Pyranoanthocyanin pigments can be produced by free anthocyanins through cycloaddition reactions or direct reactions with certain wine constituents, such as metabolites of various yeasts (e.g., acetaldehyde, vinylephenol, and pyruvic acids). Cycloaddition products known as pyranoanthocyanins are anthocyanin molecules that contain an additional pyran ring between the C4 position in the C ring and the hydroxyl group on the C5 position in the A ring. It is one of the most significant anthocyanin-derived pigments in red wine. The various reactions of the cycloaddition of free anthocyanin in red wines are summarized in Figure 4.

Pyranoanthocyanins can be produced by the reaction of free anthocyanins with both hydroxycinnamic acids and 4-vinylphenols. Vitisin A is the name given to the adduct formed between malvidin-3-glucoside and pyruvic acid. Vitisin-B is the product of the condensation of anthocyanins and acetaldehyde. In the presence of acetaldehyde, portisin is produced through the reaction between malvidin-3-glucoside-pyruvic acid derivative and (+)-catechin [32,38]. The yellow-orange coloring of the products generated and their exceptional stability, particularly in response to pH fluctuations and the action of sulfites, are the distinguishing characteristics of this reaction. In comparison to anthocyanins (λmax = 529 nm), the UV-Vis spectra of these compounds exhibit a maximum absorbance that is shifted (λmax = 503 nm and 511 nm for pyranoanthocyanins–flavanol monomers and pyranoanthocyanins–flavanol dimers, respectively) [39]. The formation of pyranoanthocyanins is likely to continue as the storage time increases in products that are rich in anthocyanins and potential reaction partners, such as wine.

#### 2.1.4. Flavan-3-ols

Flavan-3-ols are the most abundant class of polyphenols present in the grape berry and wines and are crucial for the organoleptic properties of wines, particularly astringency. They are benzopyrans, distinguished structurally by a saturated carbon chain between C2 and C3 and the absence of a carbonyl group on C4, as illustrated in Figure 1D [10].

Monomers and polymers are the two main groups of compounds within the flavan-3-ol family, with molecular weights ranging from 300 Da to 50 kDa. In the first group, (+)-catechin and its enantiomer, (−)-epicatechin, are the most prevalent monomeric forms, as are the other forms: (−)-epigallocatechin, (−)-epicatechin-3-O-gallate, and (−)-epigallocatechin-3-O-gallate. Mass spectrometry was employed by Zerbib et al. [40] to confirm the existence of monomeric flavanol hexosides in grapes and beverages. The primary monomeric flavanol hexosides found in the seed were catechin and epicatechin, and their concentrations increased as the seed matured. In the second group, flavan-3-ol oligomers and polymers include tannins or proanthocyanidins [40]. Tannins are compounds that can react with proteins and precipitate them, whereas proanthocyanidins are compounds that have the property of liberating anthocyanidins under heated acidic conditions as a result of the cleavage of the interflavanic bond [41]. It is possible to categorize proanthocyanidins into two groups based on the nature of the liberated anthocyanidins: (i) procyanidins, which are composed of (+)-catechin and (−)-epicatechin and are present in grape seeds and skins; and (ii) prodelphinidins, which are formed by (+) gallocatechin and (−)-epigallocatechin and are exclusively present in grape skin. Procyanidins are classified as dimeric, trimeric, oligomeric, and condensed. Dimeric procyanidins are dimers resulting from the condensation of two units of flavan-3-ols linked by a C4–C8 or C4–C6 bond. Trimeric procyanidins are trimers with two interflavan bonds, while oligomeric procyanidins are polymers made of three to ten flavan-3-ol units linked together. Condensed procyanidins have more than ten flavan units [41]. Studies on the structural diversity of tannins in red grapes revealed that the average size of skin tannins is substantially larger than that of seed tannins [42]. In recent pioneer investigations, the existence of tetrameric and pentameric new cyclic proanthocyanidins (also known as crown proanthocyanidins) was demonstrated for the first time in red wines from the Bordeaux region [43], while Longo et al. [44] identified a novel cyclic B-type hexameric proanthocyanidin [C90H73O36]+ in red wines.

Hydrolysable tannins are polyphenolic complexes that can be degraded into smaller fragments, primarily sugars and phenolic acids, through enzymatic or non-enzymatic hydrolysis or pH changes [10]. The central core of the structure of hydrolysable tannins is the glucose unit, which is esterified with gallic acid and gallic acid-derived moieties. The hydrolysable tannins can be categorized into two subclasses: (i) the gallotannins, which are basically linked chains of galloyl units that are typically esterified to a glucopyranose core and hydrolyzed in gallic acid; and (ii) the ellagitannins, which are composed of galloyl units esterified to a sugar core and characterized by the presence of biaryl and diaryl ether bonds between some or all of their galloyl units and hydrolyzed in ellagic acid. The aging process in oak casks is the cause of the presence of hydrolysable tannins in wines. These hydrolysable tannins originate from oak wood, while wine is aged in oak barrels as a result of the acidic pH of the medium and the alcohol present in wines. As discussed previously, these tannins are not the only source of modifications. Indeed, oxygen and micro-oxygenation are important contributors to the aging process [45,46].

Winemaking processes, such as maceration, fermentation, and aging, transfer tannins from grapes into wines. These compounds are responsible for the perception of astringency and bitterness in grapes and wines. Soares et al. recently reviewed the mechanisms of how astringency influences the overall quality of red wine [42]. Notably, the interaction between salivary enzymes and tannins is the primary established mechanism for astringency. Indeed, Sun et al. found that the astringency sensation was more noticeable when the salivary proteins were more precipitated by tannins [47]. Several parameters, including tannin concentration, tannin polymerization degree, galloylation percentage, B-ring trihydroxylation, stereochemistry of tannin subunits, and site-specific bindings, influence the intensity of astringency [48].

### 2.2. Non-Flavonoids

#### 2.2.1. Phenolic Acids

In grapes, phenolic acids are frequently divided into two main groups: hydroxybenzoic and hydroxycinnamic acids (Figure 2A,B, respectively). Mostly, the skin and pulp cells of grapes contain tartaric esters of caffeic, coumaric, and ferulic acids, which are all members of the first group of hydroxycinnamates. These acids are all characterized by a C6–C3 skeleton; however, they differ in terms of the substituents on their aromatic ring. They are accountable for the phenomenon of wine discoloration as a result of the oxidation process. Caftaric acid is the most prevalent ester in grapes, with an average concentration of 170 mg/kg. Coutaric acid and fertaric acid are the next most prevalent esters, with 20 mg/kg and 5 mg/kg, respectively [49]. These relative proportions are maintained in wine and found primarily as trans isomers, although they are also present in cis forms to a lesser extent [50]. Conversely, hydroxybenzoic acids possess a C6–C1 skeleton that is composed of an aliphatic carbon chain and a benzene ring. The most frequently encountered derivatives are vanillic, syringic, gentisic, and gallic acid. With concentrations ranging from 100 to 230 mg/kg, gallic acid is the primary component of grape purée and is present in both free and glycoside forms [50].

Gallic acid is the most prevalent hydroxybenzoic acid in wine, with a concentration ranging from 2 to 130 mg/L [33]. It is not only derived from the grape itself, but it is also formed by the hydrolysis of hydrolysable and condensed tannins (gallic acid esters of flavan-3-ols). The concentrations of hydroxybenzoic acids in wine exhibit significant variation in response to the grape variety and the growing conditions. The concentrations of hydroxybenzoic acids are found to be lower in wine than the hydroxycinnamic acids [41,51]. Red wine is reported to contain hydroxybenzoic acids at a concentration of 100 to 200 mg/L [41]. In wines, the free from of hydroxycinnamic acids are present in trace quantities. Caftaric acid (7–200 mg/L) and coutaric acid (2–20 mg/L) comprise the preponderance of phenolic acids found in wine [33]. Fertaric acid is also present, albeit in lesser concentrations. Natural hydrolysis of these esters occurs; however, esterases may enhance this process. The caftaric, p-coumaric, and fertaric esters are subsequently converted into caffeic (0.3–26 mg/L), ferulic (0.1 mg/L), and p-coumaric acids (0.4–15 mg/L) [33]. They are involved in chemical oxidation processes that result in the discoloration of grape juice and wine [52]. Their impact on the flavor of wine appears to be less significant. Conversely, the degradation of p-coumaric and ferulic acid results in the production of volatile phenols (ethyl–vinyl–guaiacol, vinyl, and ethyl phenol) that are responsible for olfactory defects [53].

#### 2.2.2. Stilbenes

Stilbenes are an additional, minor category of phenolic compounds that possess a C6–C2–C6 structure (Figure 2C). This structure is characterized by the presence of a methylene bridge that connects two benzene rings, resulting in a conjugated system. In response to Botrytis infection and other fungal attacks, vines produce the antioxidant resveratrol, which is the primary stilbene in grapes. Viniferins, which are oligomers of resveratrol, are genuine antifungal compounds. Resveratrol is available in a variety of forms, cis and trans isomers, and the glucosides of both isomers. The trans-resveratrol predominates in grapes, and it is considered the biologically active form of resveratrol. In wine, all forms of resveratrol are present; however, in grapes, cis resveratrol is absent, while trans-resveratrol and its glycosylated derivative piceid are abundant [54]. The cis/trans isomerization is facilitated by UV radiation. Red grapes, particularly skin grapes, are the primary source of resveratrol derivatives, which is why Botrytis berries have a high concentration of resveratrol [55]. In red, rose, and white wines, the cumulative levels of all forms ranged from 7 mg/L to 2 mg/L and 0.5 mg/L, respectively [56]. The hydroxylation of resveratrol and its derivative piceid results in the formation of piceatannol and its glucoside, astringin. Conversely, its oxidation induces the formation of dimers, trimers, and tetramers, including pallidol, ε-viniferin, δ-viniferin, miyabenol C, hopeaphenol, and isohopeaphenol [57].

## 3. Bioactivity of Wine Polyphenols

The ancestral use of wine as medicine for wound infection healing, diarrhea, digestion, or hemorrhoid swelling reduction has rather become in our modern time a preventive measure against non-communicable chronic diseases [58]. Indeed, in the early 1990s, the growing interests concomitant with technical advancements have led to the development of research for the study of the association between red wine consumption and health. At that time, Renaud and De Lorgeril made the observation of the French paradox from an epidemiological study [59]. Surprisingly, while coronary heart disease (CHD) was known to be positively associated with a rich-fat (saturated fat or cholesterol) diet, they, on the contrary, noticed a 40% decrease in CHD prevalence in the French study group, attributed to the protective effect of moderate red wine intake against platelet aggregation in spite of a high-fat intake. In other words, despite having a saturated fat-rich diet, French people exhibit a low incidence of CHD. This pioneering research was followed by many in vitro and in vivo animal studies that are discussed below, along with other pertinent clinical studies that have confirmed the importance of polyphenols on health without specification of gender or pre-existing conditions, as discussed below.

### 3.1. Antioxidant Effects

Oxidative stress is defined as a disruption of the equilibrium between the excessive generation and insufficient elimination of ROS and/or reactive nitrogen species (RNS), leading to an imbalance in homeostasis. The reactive metabolites, known as free radicals, specifically attack proteins and DNA, leading to significant damage to cells and tissues and initiating the onset of numerous NCDs [60,61]. In the mid-1990s, a small number of molecules with antiradical properties were identified as antioxidants. This group included 13 phenolic compounds, as first reported by Brand-Williams et al. [62] more than three decades ago. Subsequently, researchers have extensively investigated the antioxidative properties of wines, which involve the removal of harmful free radicals, inhibiting lipid oxidation, decreasing hydroperoxide generation, and neutralizing electronically excited substances [63,64]. Various techniques were utilized to assess the antioxidant capabilities of phenolic compounds isolated from different types of grapes, various grape components, and wine. These techniques are divided into chemical and in vivo methods [65]. First, among the chemical reactions involved, the chemical antioxidant capacity assays are divided into hydrogen atom transfer (HAT) and single electron transfer (ET). HAT includes the oxygen radical absorbance capacity (ORAC) assay and the ferric reducing antioxidant power (FRAP), while ET encompasses the 1,1-diphenyl-2-picrylhydrazine (DPPH) radical scavenging assay, the 2,2-azinobis(3-ethylbenzthiazoline-6-sulphonic acid) (ABTS) radical scavenging method, the N,N-Dimethyl-p-phenylene diamine (DMPD) assay, and the total radical trapping antioxidant potential (TRAP) assay. The principle of each method cited above has been reviewed by El Rayess et al. [65]. These different methods do not yield the same results since they are based on different principles and react differently with the diverse structures of wine polyphenols. However, the method from the same category yields the same tendency of results. For example, if the DPPH method indicates high antioxidant activity, the ABTS will show the same tendency. Second, in vivo techniques, such as measuring low-density lipoprotein (LDL) oxidation, are employed. Additional assays rely on the ability of wine components to hinder or influence the body’s defense mechanisms, such as enzymes like xanthine oxidase, superoxide dismutase, catalase, and glutathione peroxidase [66].

**Figure 5:**
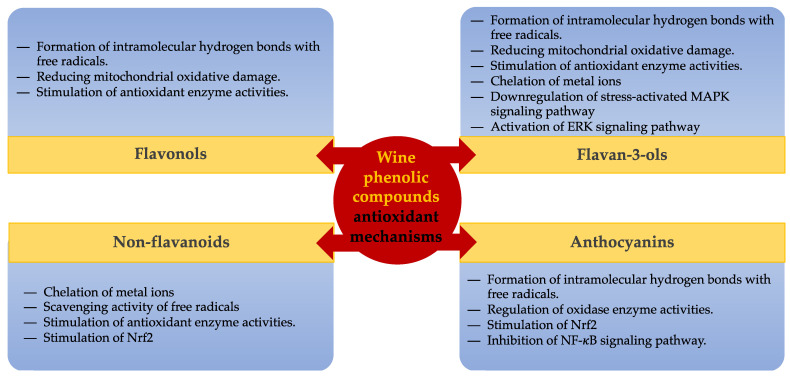
The antioxidant mechanisms of wine phenolic compounds. MAPK = mitogen-activated protein kinase; ERK = extracellular signal-regulated kinase; NF-κB = nuclear factor kappa-light-chain-enhancer of activated B cells; Nrf2 is a master regulator of the antioxidant response.

Various mechanisms have been suggested to elucidate the antioxidant properties of polyphenols. Phenolic compounds can eliminate free radicals and ROS by giving a hydrogen atom to counteract their effects, or they can also function through a process called single-electron transfer. The hydroxyl groups in phenolic compounds play a crucial role by donating hydrogen or electrons [67,68]. The second mechanism relies on the capacity of phenolic compounds to form complexes with metal ions like Fe^2+^ and Cu^2+^, thereby diminishing one of the contributors to the production of free radicals [5]. Phenolic compounds are believed to exhibit antioxidant action through the activation of antioxidant enzymes. Studies have demonstrated that phenolic compounds can activate the enzymatic antioxidant system in humans. The latter consists of a collection of enzymes, including superoxide dismutase (SOD), glutathione peroxidase (GPX), glutathione S-transferase, glutathione reductase, and catalase (CAT) [69]. The enzyme is modulated through the antioxidant-responsive element/electrophile-responsive element (ARE/EpRE). Phenolic compounds exhibit antioxidant activity by inhibiting pro-oxidant enzymes, including lipoxygenase (LOX), cyclooxygenase (COX), nitric oxide synthase (NOS), and xanthine oxidoreductase (XOR). These enzymes are responsible for the generation of ROS [5,68]. Figure 5 provides a comprehensive summary of all the mechanisms that have been cited.

### 3.2. Cardiovascular Effects

**Figure 6:**
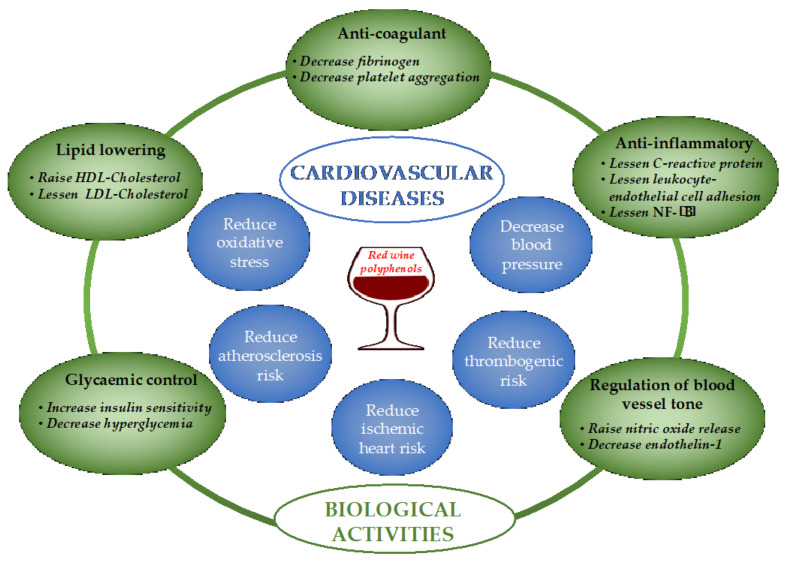
Schematic representation of the variety of biological activities of wine phenolic compounds and their potential effects on cardiovascular diseases. HDL: high-density lipoprotein, LDL: low-density lipoprotein, NF-κB: nuclear factor kappa-light-chain-enhancer of activated B cells.

Strong data supports the beneficial effects of moderate red wine consumption on cardiovascular health [70,71]. One of the initial health advantages associated with wine polyphenols was the safeguarding of cardiovascular health, commonly referred to as the “French paradox”. The observed benefits can be ascribed to the antioxidant properties, alterations in lipid profiles, anti-inflammatory effects, reduced platelet aggregation, diminished atherosclerosis, enhanced endothelial function, lowered hypertension, and increased fibrinolysis [72,73], as summarized in Figure 6. Resveratrol and flavonoids, particularly flavan-3-ols, are the most notable wine polyphenols that have demonstrated preventive benefits on cardiovascular health.

Atherosclerosis is a condition associated with inflammation in which the inner lining of blood vessels (endothelium) becomes defective and proliferative. Briefly, macrophages that are loaded with oxidized-LDL, known as foam cells, release interleukin, which leads to the migration of smooth muscle cells from the media layer to the intima of tiny arteries. Platelets aggregate at the site of injury, and a plaque called atheroma develops [74,75]. 

According to Milutinović et al., oxidized-LDL cholesterol causes inflammation, which is the initial cause of this process [76]. Polyphenol consumption significantly reduces platelet aggregation, resulting in fewer atheroma formations [77]. More specifically, consumption of wine polyphenols enhanced the blood lipid profile by increasing HDL levels and decreasing oxidized LDL. These changes are favorable for CHD [78]. Zhang et al. thoroughly reviewed quercetin, identifying it as an inhibitory regulator of inflammatory pathway markers, such as interleukins 1 and 6, vascular endothelial growth factor, and an activator of caspase 3 and nuclear factor kappa beta, responsible for all these anti-atherosclerotic effects [79].

Endothelial dysfunction in individuals with hypertension results in elevated generation of superoxide anion and hydrogen peroxide, as well as reduced synthesis of nitric oxide (NO). Consequently, this leads to vasoconstriction and an increase in blood pressure. Loke et al. and Perez-Vizcaino et al. have found that the activation of endothelial NO synthase affects the levels of vasoactive NO products in the bloodstream [80,81]. Administrating quercetin improves endothelial function. Previously, research had shown that the administration of red grape juice to patients with hypertension leads to an increase in the release of NO and a decrease in the formation of superoxide in blood vessels [82]. A more recent study found that daily consumption of 100–150 mL of whole red wine can lower post-exercise hypotension and resting blood pressure in patients with hypertension [83]. Another study demonstrated that individuals who consumed a daily dose of 200 mg of red grape cells experienced a notable reduction in blood pressure [84].

Platelet aggregation is a significant factor in the development of CHD, and its inhibition reduces the risk. In vivo experiments have shown that the administration of grape juice and red wine to rats reduces platelet aggregation. This effect is attributed to the presence of polyphenols [85]. Polyphenols primarily prevent the formation of blood clots by influencing the AA-thromboxane pathway, suppressing the creation of ROS, and decreasing the reduction of NO [86].

### 3.3. Anticancer Effects

Despite the fact that alcohol is casually linked to several cancers and is considered a carcinogen, there is evidence that moderate wine consumption may have beneficial health effects and reduce the risk of several cancers, including colon, basal cell carcinoma, lung, liver, ovarian, and prostate [87,88]. Several studies have shown that resveratrol from wine plays a special role in cancer prevention [89,90,91]. Jang et al. were the first to demonstrate this compound’s cancer-chemo preventive effect. Resveratrol inhibits the proliferation of a wide range of tumor cells, including lymphoid, myeloid, breast, prostate, stomach, colon, pancreatic, thyroid, skin, head and neck, ovarian, and cervical [92]. Resveratrol has been found to prevent cellular activities involved in the three stages of carcinogenesis: initiation, promotion, and progression. It has been shown to prevent carcinogenesis by functioning as an antioxidant, anti-inflammatory, antimutagenic, antimetastatic, antiangiogenic, antidifferentiation, antiproliferative, and pro-apoptotic agent [93]. It also regulates signal transduction, immunological response, transcription factors, growth factors, cytokines, caspases, interleukins, prostaglandin production, and cell cycle-regulating proteins [88]. 

**Figure 7:**
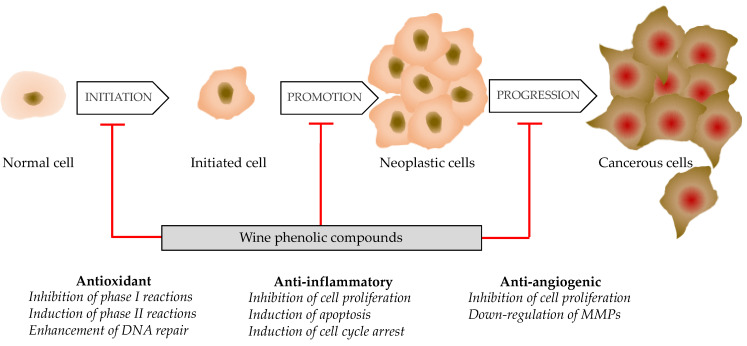
Anticancer mechanisms of wine phenolic compounds during tumor development.

The mechanisms by which wine polyphenols exert anticancer effects are summarized in Figure 7. Among them, four have been well-deciphered and include antioxidant effect, modulation of cell signaling pathways, induction of apoptosis, and suppression of metastases. 

As previously stated, wine polyphenols exhibit substantial antioxidant activity, allowing them to scavenge ROS and reduce oxidative stress-induced damage to cellular components, including DNA and proteins. Wine polyphenols aid in inhibiting tumor start by lowering oxidative stress. Wine polyphenols can influence a variety of signaling pathways that control cell growth, survival, and death. For example, resveratrol has been shown to block the Akt/mTOR pathway, which is frequently dysregulated in cancer cells, reducing cell proliferation and inducing cell death. Similarly, flavonoids like quercetin and epigallocatechin gallate (EGCG) can disrupt the MAPK and PI3K/Akt pathways, inhibiting cancer cell proliferation and inducing apoptosis [94,95].

Polyphenols can promote programmed cell death (apoptosis) in cancer cells by a variety of mechanisms, including pro-apoptotic protein activation, anti-apoptotic protein suppression, and apoptotic signaling pathway regulation. Resveratrol, quercetin, and kaempferol, for example, have been demonstrated to increase the expression of Bax and p53 while decreasing Bcl-2, resulting in caspase-mediated death in cancer cells [96].

Angiogenesis, or the development of new blood vessels, is necessary for tumor growth and metastasis. Wine polyphenols, particularly resveratrol and flavonoids, can inhibit angiogenesis by targeting key angiogenic factors like vascular endothelial growth factor (VEGF) and matrix metalloproteinase-2 (MMP-2), limiting blood supply to tumors and suppressing their growth and metastatic potential [97,98].

Metastasis is the spread of cancer cells from the initial tumor to distant locations in the body, resulting in secondary tumor growth. Wine polyphenols have been demonstrated to block numerous processes in the metastatic cascade, such as cancer cell migration, invasion, and endothelial cell adhesion. Resveratrol, for example, has been shown to lower matrix metalloproteinase (MMP) expression and impede epithelial-mesenchymal transition (EMT), reducing cancer cells’ metastatic potential [88].

### 3.4. Neuroprotection Effects

The most prevalent age-related neurodegenerative disorders include Parkinson’s disease (PD), Alzheimer’s disease (AD), Huntington’s disease, and amyotrophic lateral sclerosis (ALS). The factors leading to these diseases were reviewed by two groups of researchers [99,100]. Among these factors, oxidative stress, neuronal and glial inflammation, mitochondrial dysfunction, aggregation of amyloidogenic proteins (amyloid plaques of amyloid-beta (Aβ) peptide and intracellular neurofibrillary tangles of tau protein in AD; Lewy bodies and Lewy neuritis of intracellular amorphous α-synuclein (αS) inclusions in PD), and apoptotic pathway activation are the most commonly cited [101].

Recent literature reviews have provided evidence suggesting that consuming a moderate amount of wine may reduce the risk of developing neurodegenerative diseases [99,100,102,103,104]. In addition to ethanol, studies have indicated that non-alcoholic substances like polyphenols are associated with enhanced cognitive function [105]. Wine has polyphenols like flavonoids (flavonols, flavan-3-ols, flavanones, and anthocyanins), phenolic acids (hydroxybenzoic acids and hydroxycinnamic acids), and stilbenes (resveratrol). These polyphenols help protect the nervous system in a number of ways, such as by being antioxidants and anti-inflammatory, regulating cell signaling pathways, and increasing blood flow in the brain. These effects have been extensively examined and documented in previous studies [99,106]. Studies have proven that resveratrol effectively activates the SIRT-1 pathway, which plays a role in regulating cell cycle, inflammation, and Bcl2-mediated apoptosis. This activity helps reduce age-related neurological disorders. On the other hand, the anthocyanin components found in Cabernet Sauvignon have the ability to decrease the toxicity of β-amyloid (Aβ) peptides [107], which are biomarkers of neurodegeneration seen in AD [108]. This alternative approach to combating amyloid deposits involves the direct interaction between polyphenols found in red wine and proteins such as Tau and Aβ [109]. This discovery suggests that phenolic compounds from red wine could have therapeutic applications for AD and PD. Contrarily, researchers have found antioxidant effects in polyphenol compounds such as (−)-epicatechin, (+)-catechin, or quercetin found in Merlot or Spanish red wine [110]. These compounds work by inhibiting the Fenton reaction oxidative system and reducing the production of ROS [111]. This well-known process entailed the stimulation of the enzymes superoxide dismutase and glutathione peroxidase, as well as the enhancement of the glutathione system [110,112]. Ledo’s recent review discusses how polyphenols modulate the nitrate–nitrite–nitric oxide pathway, explaining their role in neurovascular function [113]. Collectively, these studies have shown the significance of the bioactivity of red wine polyphenols in regulating neurodegeneration.

## 4. Epidemiological Studies, Red Wine Polyphenols, and Non-Communicable Diseases

Several recent reviews have intensively summarized the impact of specific red wine polyphenols on health [102,114,115,116,117,118,119,120]. Here, we intentionally selected the most recent epidemiological findings that have highlighted red wine polyphenols as protective factors against cardiovascular and vascular diseases; neurological, inflammatory, and metabolic disorders; aging; and tumorigenesis, all mostly driven through their antioxidant and anti-inflammatory properties. 

### 4.1. Cardiovascular Diseases 

The recent work of Salazar et al. was among the first epidemiological studies to demonstrate that the higher dietary intake of flavonoids from a predominant source of red wine significantly contributed to the lower prevalence of subclinical atherosclerosis in an adult Spanish male population subgroup [121]. Another interesting recent cross-sectional study made in the United Kingdom and involving 100 female pairs of twins has shown that dietary polyphenol intake from red wine was a determinant factor for lower cardiovascular risks [122]. In spite of the small sample size, the authors nonetheless clearly demonstrated that protection from cardiovascular diseases was related to a wide range of smaller phenolic compounds called polyphenol metabolome. The most recent evidence that reinforced these findings of cardioprotective effects of polyphenols comes from a cross-sectional study conducted in Saudi Arabia on overweight and obese individuals who displayed better components of the metabolic syndrome, such as systolic blood pressure and triglyceride levels associated with dietary polyphenol intakes [123].

### 4.2. Type 2 Diabetes

Type 2 diabetes (T2D) is one of the major metabolic disorders that may arise from several co-morbidities, such as insulin resistance, obesity, and/or metabolic syndrome and others [124]. T2D is the ultimate stage of chronic impaired blood glucose management, starting with glucose intolerance followed by prediabetes. A cross-sectional study conducted from the PREDIMED-Plus cohort in Spain showed that a daily mean polyphenol intake of about 846 ± 275 mg may decrease the incidence of T2D and its related co-morbidities [125]. From the same cohort study, it was confirmed that stilbenes, mostly resveratrol, play a major role in blood glucose control, as evidenced by the improvement of glycated-hemoglobin HbA1C levels in the subjects [126].

### 4.3. Gut Health

Ulcerative colitis (UC) and Crohn’s disease are two chronic-relapsing-remitting conditions that affect the large intestine with no consensus for successful common nutritional management [127]. Taladrid et al. conducted a case-control interventional study that was the first to observe the beneficial effects of moderate and regular consumption of red wine in patients suffering UC during the acute phase of this inflammatory bowel disease [128]. A daily consumption of 250 mL of red wine for four weeks restored both oral and gut microbiota, as evidenced by the improvement of the alpha diversity observed with the Shannon–Wiener diversity index and Simpson diversity index. The significant decrease in the inflammatory marker calprotectin may explain the protective role of polyphenols against the inflammatory process. Patients reported improvement in their systemic and bowel symptoms, as well as emotional and social components of their quality of life in comparison to their control counterparts, confirming this overall. Indeed, the recent work of Li et al. [122] gave evidence that cardiovascular risk relies on microbiota composition. The specific genus 5-7N15 of the phylum *Bacteroidetes* was responsible for the transformation of dietary polyphenol into phenolic acids found in urinary metabolites that were negatively associated with Heart Score in the high polyphenol intake group. This study highlights the need to further explore the relationship between dietary polyphenols that act as prebiotics by promoting gut microbiota growth and the type of polyphenol metabolites issued from the gut microbiota that, in turn, will determine their absorption or excretion and, therefore, the beneficial effect on endothelial cells and cardiovascular risk.

### 4.4. Neurological Disorders and Aging

To the best of our knowledge, Rodrigo-Gonzalo et al. were the first to conduct a randomized clinical trial studying the effect of dietary intake of polyphenols from grapes on aging-related cognitive functions using a thorough, elaborated protocol [129]. Rodrigo-Gonzalo [130] reviewed several other studies that found the positive impact of polyphenols from diverse plant sources on cognitive performance. One of the clearest findings was that a daily intake of 50 g of red raisins for six months significantly contributed to minimizing the aging-related cognitive impairments in healthy older adults aged more than 70. This study is intriguing as it highlights the vast potential of polyphenol bioactivity on the tryptic microbiota–gut–brain axis. Because neurological diseases are known to be linked to poor microbiota composition, their results imply that the adequate dietary intake of such bioactive compounds may exert their protective effects on many neurodegenerative diseases related to aging or poor gut health.

### 4.5. Tumorigenesis and Cancer

Because of its ethanol content, red wine is a risk factor for the occurrence and development of certain types of cancer. However, studies have shown that moderate red wine intake exerts protective effects due to its content of other more beneficial polyphenol compounds, making this association controversial. Martínez-González raised the question of whether red wine must be removed from the Mediterranean diet, which encourages moderate red wine daily consumption, because it may increase the risks of breast cancer [131]. However, as recently reviewed [132], no epidemiological study on humans has confirmed the effect of red wine polyphenols on in vitro human cell models of chronic diseases, including cancer. 

These latest findings, along with others, underscore the necessity for further exploration and clarification on how the type and/or frequency of dietary intake of red wine polyphenols, along with their respective metabolites, influence biomarkers associated with NCDs. The unusually growing incidence of these debilitating diseases in the youth population urges the need to implement preventive and managerial measures through dietary lifestyle changes targeting the most beneficial source of polyphenols to reach the best results.

## 5. Conclusions

In conclusion, wine phenolic compounds represent a diverse group of bioactive molecules that contribute significantly to the sensory properties, functionality, and health benefits of wine. These compounds, primarily derived from grapes, exhibit potent antioxidant, anti-inflammatory, and cardioprotective effects, which have been linked to reduced risks of NCDs, such as cardiovascular disorders, certain cancers, and neurodegenerative conditions. The chemistry of wine phenolics is complex, involving flavonoids, phenolic acids, tannins, and stilbenes, each playing a distinct role in wine’s flavor profile and potential health-promoting properties.

The interplay between phenolic content and factors such as grape variety, terroir, winemaking techniques, and aging processes further enhances wine’s diversity, making it a unique and valuable component of human diet and culture. While moderate wine consumption, particularly red wine, is associated with these health benefits, it is essential to emphasize responsible drinking to avoid negative health effects related to excessive alcohol consumption. Continued research into wine phenolic compounds is crucial for several reasons. First, while the health benefits of these compounds, such as their antioxidant and anti-inflammatory effects, are well documented, more studies are needed to fully understand their bioavailability and metabolism in the human body. This includes determining how different phenolic compounds are absorbed, distributed, metabolized, and excreted, as well as how they interact with gut microbiota. Variability in individual responses, influenced by factors such as genetics, diet, and overall health, also warrants further investigation.

Second, the potential synergistic effects between different phenolic compounds and other components of wine or food should be explored. Understanding how these compounds work together may reveal new insights into optimizing their health benefits, both in wine and in broader dietary contexts.

Moreover, innovative winemaking techniques, yeasts, and bacteria strains, as well as agricultural practices, could enhance the phenolic content of wines, further increasing their health-promoting properties. Research into the impact of viticultural practices, grape variety selection, and environmental factors such as soil and climate (terroir) on phenolic profiles is also critical. Additionally, there is growing interest in developing non-alcoholic or low-alcohol wine alternatives that retain high phenolic content, allowing consumers to enjoy the health benefits of wine phenolics without the risks associated with alcohol.

In the broader context of public health, understanding the long-term impact of moderate wine consumption and its phenolic components on human health, especially in populations with varying dietary patterns and health conditions, is essential. These studies could inform dietary recommendations and help refine guidelines for wine consumption, maximizing health benefits on pre-existing conditions of NCDs while minimizing their risks. More research is needed to decipher the mechanism of red wine polyphenol and its potential differential effects during life span and among gender. Ultimately, continued research into wine phenolic compounds will deepen our knowledge of their role in promoting health and may lead to new applications in functional foods, nutraceuticals, and therapeutic interventions.

## Data Availability

All data are available in the main text.
